# Thymidine phosphorylase in cancer cells stimulates human endothelial cell migration and invasion by the secretion of angiogenic factors

**DOI:** 10.1038/bjc.2011.74

**Published:** 2011-03-08

**Authors:** I V Bijnsdorp, F Capriotti, F A E Kruyt, N Losekoot, M Fukushima, A W Griffioen, V L Thijssen, G J Peters

**Affiliations:** 1 Department of Medical Oncology, VU University Medical Center, PO Box 7057, 1007 MB, Amsterdam, The Netherlands; 2 Department of Medical Oncology, University Medical Center Groningen, Groningen, The Netherlands; 3 Tokushima Research Center, Taiho Pharmaceutical Co., Ltd, Tokushima, Japan; 4 Department of Radiotherapy, VU University Medical Center, Amsterdam, The Netherlands

**Keywords:** thymidine phosphorylase, thymidine phosphorylase inhibitor, angiogenesis, deoxyribose

## Abstract

**Background:**

Thymidine phosphorylase (TP) is often overexpressed in tumours and has a role in tumour aggressiveness and angiogenesis. Here, we determined whether TP increased tumour invasion and whether TP-expressing cancer cells stimulated angiogenesis.

**Methods:**

Angiogenesis was studied by exposing endothelial cells (HUVECs) to conditioned medium (CM) derived from cancer cells with high (Colo320TP1=CT-CM, RT112/TP=RT-CM) and no TP expression after which migration (wound-healing-assay) and invasion (transwell-assay) were determined. The involvement of several angiogenic factors were examined by RT–PCR, ELISA and blocking antibodies.

**Results:**

Tumour invasion was not dependent on intrinsic TP expression. The CT-CM and RT-CM stimulated HUVEC-migration and invasion by about 15 and 40%, respectively. Inhibition by 10 *μ*M TPI and 100 *μ*M L-dR, blocked migration and reduced the invasion by 50–70%. Thymidine phosphorylase activity in HUVECs was increased by CT-CM. Reverse transcription-polymerase chain reaction revealed a higher mRNA expression of bFGF (Colo320TP1), IL-8 (RT112/TP) and TNF-*α*, but not VEGF. Blocking antibodies targeting these factors decreased the migration and invasion that was induced by the CT-CM and RT-CM, except for IL-8 in CT-CM and bFGF in RT-CM.

**Conclusion:**

In our cell line panels, TP did not increase the tumour invasion, but stimulated the migration and invasion of HUVECs by two different mechanisms. Hence, TP targeting seems to provide a potential additional strategy in the field of anti-angiogenic therapy.

The platelet-derived endothelial cell growth factor (PD-ECGF) is also known as thymidine phosphorylase (TP). Numerous immunohistochemical and TP-activity studies have shown an increased TP expression and activity in a wide-range of solid tumours, compared with normal healthy tissues (Ackland and Peters, 1999; De Bruin *et al*, 2006; Bronckaers *et al*, 2009a, 2009b). A high TP expression in these tumour sites has clearly been related to a high-microvessel density, the induction of metastasis and a poor prognosis for the patient (Kidd *et al*, 2005; Nakayama *et al*, 2005; Hayashi *et al*, 2008). The location of TP expression varies between tumour type and grade and has been reported to be highly expressed in tumour cells (O’Brien *et al*, 1996), the invasive part or the tumour (Kubota *et al*, 1997; Yoshikawa *et al*, 1999; Hayashi *et al*, 2008) or in the tumour stromal cells (Nagaoka *et al*, 1998; Sakamoto *et al*, 1999; Liakakos *et al*, 2006; Ruckhäberle *et al*, 2010). From these studies about TP, we conclude that TP is involved in two different, but overlapping actions. First, it is related to a higher invasive property and metastatic potential of the cancer cells (De Bruin *et al*, 2006; Yu *et al*, 2008; Bronckaers *et al*, 2009a, 2009b). Second, it is related to an increased angiogenesis. The mechanisms underlying TP aggressiveness in cancer cells are possibly by comparable cellular events as for that of angiogenesis. However, the exact molecular mechanisms behind these actions remain unclear.

Thymidine phosphorylase catalyses the reaction of thymidine (TdR) to deoxyribose-1-phosphate (dR-1-P) and thymine. From this reaction, deoxyribose (dR) can be formed (Bijnsdorp *et al*, 2010a). It is believed that dR has a role in the angiogenic effects of TP (Hotchkiss *et al*, 2003), as dR can be secreted from the cells (Bijnsdorp *et al*, 2010a). Deoxyribose has shown angiogenic properties in various *in vitro* and *in vivo* studies (Stevenson *et al*, 1998; Hotchkiss *et al*, 2003; Sengupta *et al*, 2003). Exposure of endothelial cells to dR and TP stimulated both the migration and invasion, and activated the focal adhesion kinase (FAK) and p70/S6k (Hotchkiss *et al*, 2003; Seeliger *et al*, 2004). Focal adhesion kinase has an important role in the invasion and migration of cells and also in cell death regulation (McLean *et al*, 2005). P70/S6k is the important downstream kinase of mTOR, regulating cell proliferation, metabolism and also angiogenesis (Chiang and Abraham, 2007). In addition to angiogenesis, the mTOR-FAK signalling pathway also seems to be involved in the invasive potential of TP in cancer cells. Besides sugars that are produced by TP conversion of TdR, other mechanisms are possibly involved as well. A high TP expression has been related to an increased secretion of angiogenic factors, such as IL-8 and bFGF (Brown *et al*, 2000). Moreover, TP is often co-expressed with the important angiogenic factor vascular endothelial growth factor (VEGF) (Sivridis *et al*, 2002). However, whether TP can regulate VEGF is unclear.

To reduce tumour aggressiveness and angiogenesis, TP inhibitors have been synthesised. The TPI is a very potent and specific inhibitor of TP and does not inhibit uridine phosphorylase (UP) (Fukushima *et al*, 2000). Uridine phosphorylase can also break down thymidine in cancer cells (Temmink *et al*, 2007). Another possibility to inhibit one of the downstream biological actions of TP is by addition of L-deoxyribose, a stereoisomer of dR (Nakajima *et al*, 2004). Although L-dR is often used to study TP functioning, dR is not the only product that is responsible for the pro-angiogenic activity of TP (Brown and Bicknell, 1998; Brown *et al*, 2000). Therefore, the use of specific TP inhibitors are evaluated for their anti-angiogenic potential in cancer cells.

It is not exactly known how cancer cells with a high TP expression can stimulate endothelial cells to form new blood vessels. The aim of the present study was to evaluate the role of TP in cancer cells on the proliferation, migration and invasion of endothelial cells and to identify several potential angiogenic factors in their involvement in these process. Therefore, we used conditioned medium from TP-expressing cells to determine whether tumour cells secrete molecules that stimulate angiogenesis. As the TP expression in cancer cells also increase their aggressiveness, we also determined the level of invasion and intracellular signalling of colon cancer and bladder cancer cell lines, with and without TP expression.

## Materials and methods

### Cell lines

Human colon cancer cell lines Colo320, Colo320TP1 (TP-transfected; De Bruin *et al*, 2003), RT112 and RT112/TP (TP-transfected; Brown *et al*, 2000) were cultured as monolayers in DMEM, supplemented with 20 mM Hepes and 10% fetal calf serum (FCS). Human umbilical vein endothelial cells (HUVECs) were isolated from human umbilical cords and were cultured in M199 medium, supplemented with 10% FCS (PAA laboratory GmbH, Cölbe, Germany), 10% human serum (HS) and ECGF. All cells were maintained in a humidified 5% CO_2_ atmosphere at 37°C. For all experiments in which HUVECs were used, at least three different isolations (e.g. donors) were examined for their (angiogenic) response.

### TP enzymatic activity

Thymidine phosphorylase enzymatic activity was performed by HPLC measurement of TdR conversion to thymine as described previously (De Bruin *et al*, 2004). In brief, HUVECs were exposed to the CM with 100 *μ*M TdR for 6 h, after which the medium was collected. For each added TdR concentration in the CM, a *t*=0 timepoint was collected and TdR levels were measured in these samples for correction. In addition, in the CM, the TP activity was measured after 24 and 48 h incubation with 100 *μ*M TdR incubation at 37°C. Subsequently, trichloroacetic acid (TCA) was added for 20 min and samples were centrifuged at 14 000 **g** at 4°C for 10 min. The supernatant was transferred to a new vial, and the pH was neutralised. Thymidine, deoxyribose and thymine were measured by HPLC analysis for nucleosides with UV detection as described previously (Laurensse *et al*, 1988).

### Concentrating conditioned medium

Cancer cells were seeded (2–2.5 × 10^6^ cells/T75 culture flask (Greiner Bio-One, Alphen a/d Rijn, The Netherlands) in exactly 15 ml medium. After 3 days of normal growth, the medium was collected and filtered through a 0.22 *μ*m filter to remove any floating cells. Subsequently, the medium was concentrated 20 × using Amicon Ultra 15 Centrifugal filters (Milipore, Billerica, MA, USA). This concentrated medium was stored at −20°C in aliquots. For each experiment, the concentrated medium was thawed and diluted 1 : 20 in HUVEC-medium. From this diluted medium, the conditioned medium (CM) is referred as C-CM (Colo320), CT-CM (Colo320 TP1), R-CM (RT112) and RT-CM (RT112/TP).

### Migration assay

Human umbilical vein endothelial cell migration was determined using the wound healing assay as described previously (Bijman *et al*, 2006; Bijnsdorp *et al*, 2010b). Human umbilical vein endothelial cells were seeded (100 000 cells per well) in duplicates in 1% gelatin coated wells of a 24 wells plate (Corning, Schiphol, The Netherland). Cells were grown till confluence and a scratch wound was applied in two perpendicular directions with a sterile pipet tip. Subsequently, cells were washed two times with HBSS and cells were exposed to the various conditioned media, diluted in HUVEC-medium with 5% FCS and 5% HS. When blocking antibodies were used, the medium was 30 min pre-incubated in the diluted conditioned medium. Wounds were captured at × 2.5 magnification with a microscope (TCS 4D, Leica, Jena, Germany), and Q500MC software (Leica) at time points 0, 3 and 6 h. At all indicated time points, the wound width was measured in four areas and compared with the initial width at the 0 h time point and were corrected for the negative control (set at 0%).

### Invasion assay

The invasion assay was carried out using transwell chambers with a fluorescence-blocking 8 *μ*m pore filter insert (#35–1152; HTS Fluoroblock Insert, Falcon, Becton Dickinson Labware, Bedford, MA, USA) (Bijman *et al*, 2006). The insert was coated overnight at room temperature (RT) with 100 *μ*l matrigel (50 ng ml^−1^ in PBS; Sigma-Aldrich Chemicals, Zwijndrecht, The Netherlands). For HUVEC invasion, the bottom compartment was coated with 1% gelatin. Cells (50 000/insert) were seeded in medium with 1% FCS and 1% HS without ECGF. In the bottom compartment, the CM diluted in HUVEC-medium with 1% FCS and 1% HS was added. Human umbilical vein endothelial cells were allowed to invade for 8 h. The level of invasion was set relative to the positive control and normalised to the negative control. Cancer cells (20 0000/insert) were seeded in serum free medium. In the bottom compartment, medium with 10% serum was added as chemoattractant. Cells were allowed to invade for 18 h. For both HUVECs and cancer cells, 5 *μ*M calcein-AM was added 30 min before analysis to the lower compartment and fluorescently labelled cells were counted.

### Endothelial cells proliferation

At 24 h after seeding HUVECs (100 000 cells/T25 flask (Greiner Bio-One), cells were exposed to CM diluted in HUVEC medium containing 5% FCS and 5% HS. Cell numbers were counted after 3 and 6 days exposure using a counting chamber (Bürker-Türk, Paul Marienfeld GmbH & Co. KG, Lauda-Königshofen, Germany).

### Western blotting

Cells were exposed to various conditions, as indicated. After exposure, cells were washed twice with ice-cold PBS and lysed in lysis buffer (Cell Signaling Technology Inc., Danvers, MA, USA). Cell lysates were scraped, transferred into a vial and centrifuged at 11 000 **g** at 4°C for 10 min. Supernatants were transferred to a new vial and protein amounts were determined by the Bio-Rad assay, according to the manufacturer's instruction (Bio-Rad Laboratories, Veenendaal, The Netherlands). From each condition, 30 *μ*g of protein was separated on a 8–12% SDS–PAGE and electroblotted onto polyvinylidenedifluoride (PVDF) membranes (Millipore Immobilon –FL PVDF, 0.45 *μ*m). Subsequently, the membranes were blocked for 1 h at room temperature (RT) in Odyssey blocking buffer (Odyssey blocking buffer #927–40003, Westburg, Leusden, The Netherlands) and incubated overnight at 4°C with the primary antibodies (Anti-focal adhesion kinase, anti-Akt, anti-MAPK, anti-p70/S6k antibodies) were purchased from Cell Signaling Technology Inc., dilution 1 : 1000–10 000 in Odyssey blocking buffer 1 : 1 diluted with PBS-T (PBS with 0.05% Tween-20). The membrane was washed five times in PBS-T and incubated with the secondary antibody (1 : 10 000 goat-*α*-mouse-IRDye (800CW; #926–32210 and 680; #926–32220) or goat-*α*-rabbit-IRDye (800CW; 926–32211 and 680; #926–32221), Westburg) for 1 h at RT in the dark. After incubation, the membrane was washed in PBS-T and followed by 5 min washing in PBS without Tween-20 to decrease the background signal. Subsequently, the bands were scanned using an Odyssey Infrared Imager (Westburg), 84 *μ*m resolution, 0 mm offset and with high quality (Mathews *et al*, 2009).

### RT–PCR

Reverse transcription-polymerase chain reaction analysis of the angiogenic factors VEGF-A, bFGF, PLGF, TNF-*α* and IL-8 was performed as described previously. (Thijssen *et al*, 2004, 2008) In brief, Colo320, Colo320 TP1, RT112 and RT112/TP cells were cultured for 3 days, after which cells were collected and RNA was isolated. Targets were normalised to HPRT.

### VEGF, IL-8 and TNF-α detection

Vascular endothelial growth factor secretion was determined using a Quantikine human VEGF immunoassay ELISA (# DVE00; R&D systems Inc., Minneapolis, MN, USA), IL-8 by a human IL-8 ELISA kit (Becton Dickinson, Breda, The Netherlands) and TNF-*α* Sanquin PeliKine human TNF-*α* ELISA kit (#M1920). The ELISAs were performed according to manufacturer's instructions. In brief, concentrated medium was examined undiluted or diluted 20 × and 200 × . Subsequently, VEGF, IL-8 and TNF-*α* concentrations were determined and calculated in relation to the calibration curve.

### Statistical analysis

For calculating significant differences between the parental and the transfected cells or between treated and untreated samples, the two-tailed paired Student's *t*-test was used. The values were considered significantly different when *P*<0.05.

## Results

### The role of TP in cancer cell invasion

**Figure 1:**
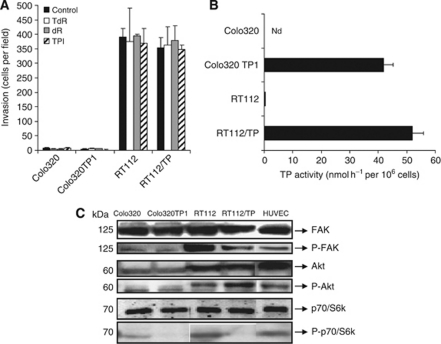
Role of TP in cancer cells. (**A**) Invasion capacity of the various cell lines after 24 h of invasion through an extracellular matrix (50 ng ml^−1^ matrigel) using the transwell invasion assay. Values are means of at least three independent experiments ±s.e.m. (**B**) Thymidine phosphorylase enzymatic activity of the various cancer cells. Colo320 had no detectable TP activity. Values are means of at least three independent experiments ±s.e.m. (**C**) Western blot of cancer cell lines expression of the kinases (phosphorylated) FAK, Akt and p70/S6k in Colo320, Colo320 TP1, RT112, RT112/TP and in HUVEC cells.

To determine whether TP expression in cancer cells results in an increased aggressiveness, Colo320, RT112 cells and their TP-transfected variants were examined on their invasion capacity. Colo320 and Colo320 TP1 cells hardly invaded, while RT112 and RT112/TP cells had a high invasion capacity (Figure 1A). Colo320 and RT112 had no TP activity, while their transfected variants had comparable TP activity (Figure 1B). There was no relation between the level of intrinsic TP expression and level of invasion. Furthermore, the invasive potential was not increased by stimulating TP activity with TdR or by the main secreted product of this reaction dR (Figure 1A). The TP inhibitor, TPI (10 *μ*M), did not affect the invasion of these cancer cell lines. Taken together, these data suggest that intrinsic TP expression in RT112/TP and Colo320 TP1 cancer cells does not affect their invasive characteristic.

### Expression levels of phospho-FAK and phospho-p70/S6k were lower in TP-expressing cells

To determine whether differences in invasion between the two cell types were related to a different expression level of the FAK-Akt-p70/S6k pathway, the expression and phosphorylation levels were determined by western blotting (Figure 1C). All cell lines constitutively expressed FAK and Akt and p70/S6k. Phosphorylation levels of FAK and Akt were higher in RT112 and RT112/TP. Interestingly, compared with the parental cell lines, phosphorylated-FAK and phosphorylated-p70/S6k were observed to be lower in the TP-transfected variants, although the invasion levels were not different between the cell lines. Taken together, the phosphorylation levels of the kinases in these cell lines were different when TP was highly expressed, which might be related to the difference in their behaviour.

### CM from cancer cells with a high TP expression does not stimulate endothelial cell proliferation

To determine the angiogenic response of endothelial cells, various angiogenic properties were studied using endothelial cells, including proliferation, migration and invasion. As angiogenesis is a process that involves endothelial cells, we examined the angiogenic responses in endothelial cells derived from human umbilical veins (HUVECs). We prepared conditioned medium from the cancer cells with high TP and no TP expression and stimulated HUVECs with this medium and subsequently examined the angiogenic responses. To determine whether cancer cells with a high TP expression can stimulate HUVEC proliferation, HUVECs were exposed to the CM of the cancer cells and HUVECs were counted after 3 and 6 days of culture. Under these used conditions (5% of both FCS and HS), the CM did not stimulate the proliferation of the HUVECs at all (data not shown). This indicates that cancer cells did not secrete products that directly stimulated HUVEC proliferation.

### CM from cancer cells with a high TP expression can stimulate endothelial cell migration

**Figure 2:**
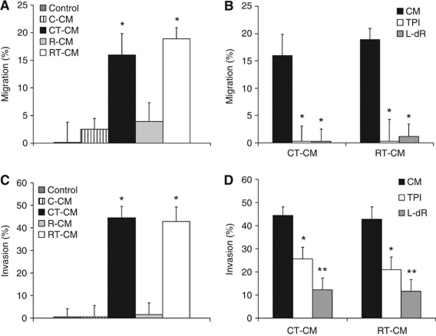
Migration and invasion of HUVECs. (**A**) Migration after 6 h stimulation with R-CM, R/TP-CM, C-CM or C/TP-CM. Endothelial cell (EC) medium is the medium in which the concentrated medium was diluted 20 × . Values represent means of at least five independent experiments ±s.e.m. Significant differences between the CM and EC medium are indicated in the graph ^*^P<0.01. (**B**) Migration after 6 h stimulation of HUVECs with the CM with and without 10 *μ*M TPI and 100 *μ*M L-dR. All values represent means of at least 3–5 independent experiments ±s.e.m. Significant differences between CM alone and CM plus inhibitor (^*^P<0.01) are indicated in the graph. (**C**) Invasion stimulation after 8 h stimulation with R-CM, R/TP-CM, C-CM or C/TP-CM. Endothelial cell medium is the medium in which the concentrated medium was diluted 20 × . Values represent means of at least five independent experiments ±s.e.m. Significant differences between the CM and EC medium are indicated in the graph ^*^P<0.005. (**D**) Invasion after exposure of HUVECs with the CM with and without 10 *μ*M TPI and 100 *μ*M L-dR. All values represent means of at least 3–5 independent experiments ±s.e.m. Significant differences between CM alone and CM plus inhibitor (^*^P<0.05, ^**^P<0.005) are indicated in the graph.

To determine whether the CM derived from cancer cells with a high TP expression could stimulate the migration of the HUVECs, the wound-healing assay was performed. We used experimental conditions comparable with the proliferation assay, because under these conditions the HUVECs were viable, but do not proliferate. The CM derived from TP-expressing cells (RT-CM and CT-CM) stimulated the migration by about 15–20%, respectively. The CM derived from non-TP-expressing cells (C-CM and R-CM) did not significantly increase the migration of the HUVECs (Figure 2A). Addition of 10 *μ*M TPI and 100 *μ*M L-dR to RT-CM and CT-CM reduced HUVEC migration back to control levels (Figure 2B), while these inhibitors did not affect the migration of the controls or the C-CM and R-CM (data not shown).

### CM from cancer cells with a high TP expression stimulate endothelial cell invasion

To determine whether the CM derived from cells with a high TP expression can increase the invasion capacity of the HUVECs, the transwell invasion assay was performed. In agreement with the wound-healing assay, the CM derived from cells with a high TP expression attracted endothelial cells by about 40–45% (Figure 2C). The CM from cells with no TP expression did not significantly increase the invasion of HUVECs (Figure 2C). The TPI (10 *μ*M) reduced the invasion that was induced by RT-CM and CT-CM by almost 50% (Figure 2D), which may be related to the addition of TPI to the conditioned medium and not to the cancer cells during medium collection. The L-dR inhibited the RT-CM and CT-CM induced invasion by almost 90%. The TPI and L-dR did not inhibit or stimulate the invasion of the controls (data not shown).

### Activation of p70/S6k expression levels of endothelial cells

**Figure 3:**
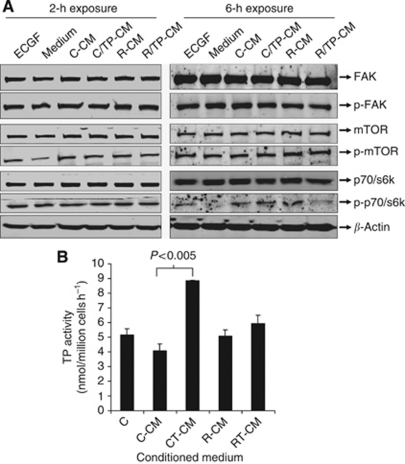
(**A**) Western blot of (phosphorylated) kinases after 2 and 6 h stimulation of HUVECs with the CM. As a positive control, complete medium with ECGF was used (ECGF). As negative control (medium), medium in which the concentrated medium was diluted was used (1% FCS and 1% HS). The *β*-actin was used as a protein loading control. (**B**) Human umbilical vein endothelial cell TP enzymatic activity after stimulation with the various indicated CM. All values represent means of at least three independent experiments ±s.e.m. Significant differences between CM and control (C) medium are indicated in the graph.

The differences in migration and invasion between HUVECs stimulated by the CM from TP-expressing cells and non-TP-expressing cells may be related to the activation of different intracellular signalling pathways. To study this, a western blot was performed determining expression levels of (phosphorylated) FAK, Akt and p70/S6k. These kinases were previously reported to be involved in TP-mediated cell migration and invasion (Figure 3A). Upon 2 h stimulation of endothelial cells with the control medium, phosphorylated levels of mTOR were decreased. After 6 h, these levels were back to control levels and phosphorylation levels of the downstream p70/S6k were decreased. The CM resulted in increased phosphorylation levels of mTOR after 2 h. After 6 h, exposure to C-CM, CT-CM and R-CM resulted in increased p-p70/S6k phosphorylation levels. Phosphorylated levels of mTOR and FAK were not changed at all. This indicates that these signalling molecules were probably not involved in the migration or invasion induced by the CM derived from TP-expressing cells.

### TP enzymatic activity in HUVECs was increased by the CM from Colo320 TP1 cells

To determine whether the effect of the CM was related to an induction of TP expression of the HUVECs, TP enzymatic activity was determined. The C-CM and R-CM did not increase TP activity. CT-CM increased the TP activity of the HUVECs, compared with the C-CM (Figure 3B). However, RT-CM only slightly increased the TP activity of the HUVECs. As TP can be secreted from the cells and thus be present in the conditioned medium, the TP activity was evaluated in the CM. Thymidine phosphorylase activity in this CM was lower than 10 pmol/million seeded cells h^−1^ in all the conditioned media, thus the TP present in this CM could be neglected. The increase in invasion and migration induced by the CT-CM is possibly related to the increased TP expression of the HUVECs, and is an explanation why TPI (partially) inhibited the migration and invasion capacity of these cells.

### Increased expression of IL-8, TNF-*α* and bFGF in TP-expressing cells

**Figure 4:**
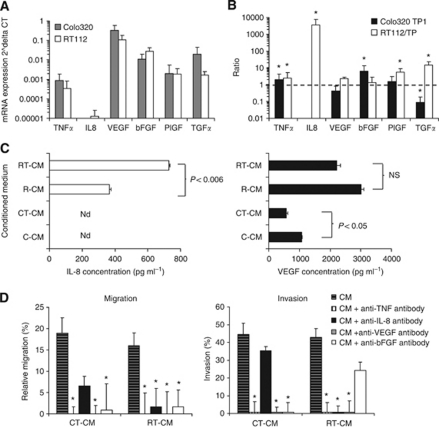
Effect of TP expression of angiogenic factors and their effect on migration and invasion. (**A**) The mRNA expression levels of angiogenic factors in cancer cells by RT–PCR. Values represent means ±s.e.m. (**B**) The mRNA expression ratios between parental and TP transfected cell lines. Values represent means ±s.e.m. Significant differences between parental and TP transfected cells are indicated in the graph ^*^P<0.05. (**C**) Interleukin-8 (IL-8) and VEGF concentration in the conditioned medium as determined by ELISA. Values represent means of at least three independent experiments ±s.e.m. Significant differences between the CM derived from TP-transfected cells and the parental cells are indicated in the graph. (**D**) Inhibition of migration and invasion by blocking antibodies of the differentially expressed angiogenic factors. The IL-8, bFGF and TNF-*α* blocking antibodies were used at a concentration of 50 ng ml^−1^, and avastin was used at a concentration of 100 ng ml^−1^. As a control, IgG antibodies were used, which did not inhibit the migration or invasion at all (data not shown). Values represent means of at least five independent experiments ±s.e.m. Significant differences are indicated in the graph ^*^P<0.05.

The influence of the TP-expressing cells on the increased migration and invasion of the HUVECs can be related to an increased expression and secretion of angiogenic factors. Therefore, we examined the mRNA expression levels of various important angiogenic factors in Colo320, Colo320 TP1, RT112 and RT112/TP cells (Figure 4A) (Thijssen *et al*, 2004). The most prominent difference between RT112 and RT112/TP cells was the expression of IL-8, which was much higher in RT112/TP cells (Figure 4B). However, in both Colo320 and Colo320 TP1 cells, IL-8 was not expressed. Other factors that were significantly increased include TNF-*α*, and bFGF, which were about two- to six-fold increased (Figure 4B). The differences in mRNA expression levels between Colo320 and Colo320 TP1 cells were less compared with the differences in RT112 and RT112/TP cells. Vascular endothelial growth factor was not differentially expressed between the parental and transfected cell lines. TNF-*α* was expressed to a significant lower extent in Colo320 TP1 compared with Colo320 cells, and was higher expressed in RT112/TP than in RT112 cells. Taken together, various angiogenic factors were differentially expressed in TP-expressing cells, compared with non-TP-expressing cells, indicating a role for TP in modulating the expression of these angiogenic factors. The expression of these angiogenic factors were cell-type dependent, indicating that TP is not the only factor that is involved.

To examine whether angiogenic factors were secreted, we selected VEGF, IL-8 and TNF-*α* for further investigation, as they are often implicated in stimulation of angiogenesis. TNF-*α* was hardly detectable in the conditioned medium or in the medium with ECGF (data not shown). Vascular endothelial growth factor was secreted at higher levels in C-CM than in CT-CM. The R-CM had higher levels of VEGF than the C-CM, but the levels were not different in the transfected variant (Figure 4C). In RT-CM, the IL-8 levels were much higher, compared with the R-CM, which is in agreement with the mRNA expression levels (Figure 4C). In both C-CM and CT-CM, IL-8 was not detectable.

### Inhibition of IL-8, bFGF, TNF-*α* and VEGF reduced the migration and invasion

To determine whether TNF-*α*, VEGF, IL-8 and bFGF were related to the increased migration and invasion of the HUVECs, blocking antibodies were used that specifically inhibit their angiogenic modulation (Figure 4D). The induced migration by RT-CM was completely inhibited by blocking TNF-*α*, VEGF, IL-8 and bFGF. The induced migration and invasion by CT-CM were completely blocked by antibodies against TNF-*α*, VEGF and bFGF, but not by that against IL-8. This is in agreement with the lack of IL-8 mRNA expression and absence of protein secretion. Taken together, several angiogenic factors were involved in the TP-mediated modulation of the migration and invasion of HUVECs. The blocking antibodies against VEGF, IL-8 and bFGF inhibited the migration and invasion of R-CM to some extent, although to a lesser extent than that of RT-CM (data not shown). C-CM-induced migration and invasion were not inhibited by any of the tested blocking antibodies. This indicates that IL-8, bFGF and possibly TNF-*α* and VEGF were involved in TP-induced migration and invasion.

## Discussion

The present data show that conditioned medium derived from cells with a high-TP expression, stimulate the migratory and invasive capacity of endothelial cells, by activating two different mechanisms. The increased TP expression in cancer cells stimulate the secretion of several angiogenic factors and also Colo320 TP1 medium increased the TP activity in HUVECs. The secretion of angiogenic factors was different between the two cell types. In cancer cells, a high TP expression (e.g., cancer cells transfected with the *TP* gene) was not related to an increased invasion of the cells themselves, indicating that the invasive potential in our cell lines was not related to TP expression.

Limited studies related TP to angiogenesis (De Bruin *et al*, 2006; Bronckaers *et al*, 2009a, 2009b). From these studies, it was concluded that TP expression and activity was clearly related to the presence of angiogenesis. However, the mechanism behind angiogenesis that is induced by TP-expressing cells is not completely understood. To study the angiogenic role of TP, exogenous TP or dR have been used (Brown *et al*, 2000; Hotchkiss *et al*, 2003; Seeliger *et al*, 2004). However, other factors may also be involved (Brown *et al*, 2000). Therefore, we used conditioned medium from TP-expressing cells to determine whether tumour cells secrete pro-angiogenic molecules. TP-expressing cells indeed secreted angiogenic factors (IL-8, bFGF) that stimulated endothelial cell migration and invasion, but not proliferation. The secretion of the angiogenic factors VEGF and IL-8 has been demonstrated before in RT112/TP cells by Brown *et al* (2000). Our data are in agreement with their data, indicating that IL-8 has a role in TP-mediated migration and invasion in this cell line. However, we did not find an increased VEGF secretion by RT112/TP cells, possibly because of the lower glucose levels (5.5 mM instead of standard 25 mM in culturing conditions) that Brown *et al* (2000) used in their study. The limited or even absent effect of the secreted VEGF in stimulating migration and invasion in our study is in agreement with observations from Hotchkiss, who reported that TP and VEGF are independent expressors (Hotchkiss *et al*, 2003). In addition to IL-8, we demonstrate a possible role of bFGF and TNF-*α* in TP-mediated stimulation of migration and invasion. The bFGF and TNF-*α* are well documented angiogenic growth factors that stimulate endothelial cell migration (Yoshida *et al*, 1997; Rosenkilde and Schwartz, 2004; Wang and Lin, 2008; Szekanecz *et al*, 2009). Many angiogenic factors enhance each other's effect to stimulate angiogenesis (Yoshida *et al*, 1997; Lázár-Molnár *et al*, 2000). However, inhibition of only one of these factors (even when not upregulated) can already result in a complete inhibition of the angiogenic effects, as inhibition of one of these factors (such as VEGF, which was not upregulated) resulted in a complete inhibition of migration and invasion. Therefore, a combination of these angiogenic factors could still be responsible for the angiogenic effect of TP. Future experiments should elucidate to which extent TPI affects the secretion of angiogenic factors by the tumour cells.

To study the role of TP in angiogenesis, L-dR is often used (Nakajima *et al*, 2009), including other inhibitors of TP (Liekens *et al*, 2007; Bronckaers *et al*, 2009a, 2009b). Enzymatic inhibition of TP, inhibited the induction of angiogenesis, indicating that the enzymatic activity of TP in indispensable for these effects. L-dR inhibited the migration and invasion of endothelial cells (Uchimiya *et al*, 2002), which indicates that dR produced by the cells can be one of the key factors in angiogenesis. Moreover, L-dR inhibited the formation of focal contacts between endothelial cells (Hotchkiss *et al*, 2003). In the present study, we demonstrate that Colo320TP1 cancer cells increased the TP expression of HUVECs, which might increase their angiogenic potential. The inhibitors TPI and L-dR reversed these effects, indicating that TP and dR have a role. The development of TP inhibitors such as TPI provides potential for inhibiting migration and invasion of endothelial cells, and can be combined with various anti-cancer agents.

The molecular pathway that is activated upon stimulation of TP can be different between endothelial and cancer cells. In endothelial cells, exogenously added TP and dR activate integrins, subsequent downstream signalling pathways involving FAK and p70/S6k and the migration and invasion of endothelial cells (Hotchkiss *et al*, 2003; Seeliger *et al*, 2004; Pula *et al*, 2010). FAK is a non-receptor tyrosine kinase that has a role in cell migration and cell death (McLean *et al*, 2005). However, conditioned medium only activated p70S6k, but not FAK, while the effects were not different between TP-expressing cells and the non-TP-expressing cells. Therefore, this molecular pathway may not be involved in TP-dependent migration and invasion. Apparently, exogenous TP or dR inflicts other cellular responses than cells with intrinsically high TP expression or stimulating endothelial cells with conditioned medium from TP-expressing cancer cells.

Thymidine phosphorylase expression in cancer cells might also influence their aggressiveness (Nakajima *et al*, 2004, 2006). Our data show that intrinsic TP expression of Colo320, Colo320TP1, RT112 and RT112/TP cancer cells did not increase the invasiveness of caner cells themselves. Yu *et al* (2008) reported that gastric cancer cells had a higher invasive potential and activated FAK and p70/S6k phosphorylation levels. The difference between their data and our data is possibly related to the different tumour types. Other cell types may respond different to their TP expression, possibly because of different genetic profile (De Bruin *et al*, 2006; Bronckaers *et al*, 2009a, 2009b). Possibly, TP is not a key factor in the increased invasive potential of colorectal and bladder cancer cells, but other factors need to be differentially expressed as well. Future studies should investigate these co-factors important for the invasive potential of cancer cells with a high TP expression.

In conclusion, TP can stimulate endothelial cell migration and invasion, possibly by inducing the secretion of a combination of the angiogenic factors IL-8, bFGF and TNF-*α* and increasing endothelial cell TP activity. This underlines the important potential for developing TP inhibitors as anti-angiogenesis therapy. In addition, many anti-cancer agents can increase the TP expression of cancer cells. Therefore, combining TP inhibitors with these anticancer agents can have potential for targeting cancer dually.
